# Association of dietary index for gut microbiota with frailty in middle-aged and older Americans: a cross-sectional study and mediation analysis

**DOI:** 10.3389/fnut.2025.1615386

**Published:** 2025-06-25

**Authors:** Xiaodan Li, Ying Liu, Chuyi Shen, Changzhuan Shao, Hongke Jiang

**Affiliations:** ^1^Faculty of Humanities and Social Sciences, Macau Polytechnic University, Macau, China; ^2^Vocational and Technical College, Shanghai Jian Qiao University, Shanghai, China; ^3^Faculty of Applied Sciences, Macao Polytechnic University, Macao, China; ^4^Department of Physical Education, Shanghai Maritime University, Shanghai, China

**Keywords:** frailty, gut microbiome, DI-GM, nutritional strategies, mediation effect, geriatric care

## Abstract

**Background:**

While frailty poses significant challenges in aging populations, evidence regarding gut microbiome-targeted nutritional strategies remains limited. The novel Dietary Index for Gut Microbiota (DI-GM), quantifying microbial-pertinent dietary patterns, provides a framework for personalized frailty mitigation.

**Methods:**

This cross-sectional study analyzed 12,914 middle-aged and older Americans (≥45 years) from National Health and Nutrition Examination Survey 2005–2018. Frailty status was assessed using a validated 49-item index (non-frail ≤0.21; frail >0.21). The DI-GM (range 0–10) was derived from 14 microbiota-linked dietary components through two 24-h recall interviews. Multivariable logistic regression with sensitivity analyses, restricted cubic splines (RCS), stratified analyses, and mediation effect were implemented to systematically evaluate DI-GM-frailty associations.

**Results:**

Adjusted analyses revealed a dose-dependent inverse association between DI-GM and frailty (OR = 0.86, 95%CI: 0.84–0.88 per unit increase). RCS identified a nonlinear inflection at DI-GM = 4.082 (*p* for nonlinearity = 0.031), beyond which each unit elevation corresponded to 12.6% reduced frailty probability (OR = 0.874, 95%CI: 0.806–0.947). Stratified analyses demonstrated enhanced protective effects in women (OR = 0.84 vs. male 0.90; *p* for interaction = 0.011) and college-educated individuals (OR = 0.85 vs. 0.93; *p* for interaction = 0.031). Mediation analysis indicated BMI mediated 38.28% (95%CI: 26.62–62.6, *p* < 0.001) of the DI-GM effect on frailty progression.

**Conclusion:**

Higher DI-GM scores associate with lower frailty prevalence, particularly among women and highly-educated populations, partially mediated through BMI modulation. These findings establish DI-GM as a microbiota-targeted nutritional guideline for age-related frailty interventions, bridging dietary patterns with microbial homeostasis in geriatric care.

## 1 Introduction

Frailty is a prevalent condition in older adults, characterized by a progressive decline in physiological function, diminished resilience, and heightened risks of adverse outcomes, including mortality, falls, hospitalizations, and disabilities (1, 2). The increasing global prevalence of frailty, driven by population aging, imposes substantial burdens on healthcare systems and society (3, 4).

Among modifiable risk factors in aging, the gut microbiota has appeared as a key focus, with evidence highlighting its role as a central mediator of systemic health. Nutritional approaches emphasizing fiber and fermented foods demonstrate cost-effective potential to restore microbial balance (2), counteracting chronic inflammation and metabolic dysregulation underlying frailty (5). Frailty progression is driven by interconnected mechanisms involving intestinal barrier compromise, chronic inflammation, and microbial imbalance (6–9). Targeted dietary modifications that enhance the production of anti-inflammatory metabolites may disrupt this pathological cycle (10, 11).

The development of the Dietary Index for Gut Microbiota (DI-GM) provides a methodological framework to operationalize dietary interventions, specifically designed to evaluate longitudinal relationships between habitual dietary patterns and gut microbial community trajectories (12). Higher DI-GM scores, reflecting diets rich in prebiotics (e.g., whole grains, legumes) and fermented foods (e.g., yogurt, kimchi), have been associated with reduced risks of depression (13), suicide (14), stroke (15), kidney stones (16), infertility (17), and metabolic disorders (18–20). Despite evidence linking DI-GM to chronic diseases, its association with frailty—a multidimensional aging syndrome—remains unexplored.

Therefore, this study leverages data derived from the National Health and Nutrition Examination Survey (NHANES, 2005–2018), to investigate diet-related exposures in frailty prevalence, aiming to develop microbiota-targeted dietary interventions for mitigating frailty in elder adults.

## 2 Materials and methods

### 2.1 Data acquisition

We analyzed seven cycles (2005–2018) of the NHANES, a nationally representative study coordinated by the Centers for Disease Control and Prevention using stratified multistage sampling to capture non-institutionalized Middle-Aged and Older Americans. The initial cohort comprised 70,190 participants. The analysis excluded participants meeting the following predefined exclusion criteria: (1) age under 45 years (*n* = 47,268), (2) absence of DI-GM data (*n* = 2,598), (3) incomplete frailty assessments (defined as < 80% completion of frailty-related items; *n* = 6,029), and (4) missing covariate information (*n* = 1,381). A total of 57,276 individuals were excluded based on these criteria. After excluding ineligible cases, 12,914 participants were deemed eligible and incorporated in the final analysis. Furthermore, to evaluate potential selection biases, the investigation employed systematically curated exclusion metrics for intergroup comparative assessment of enrollment-discontinuation demographic variances. Given the study’s prioritization of frailty assessment individuals aged ≥45 years, comparative analyses were conducted between the definitively enrolled cohort and age-eligible excluded counterparts (*n* = 10,008). The excluded group’s population characteristics are methodically tabulated in Supplementary Table S1.

**Figure 1:**
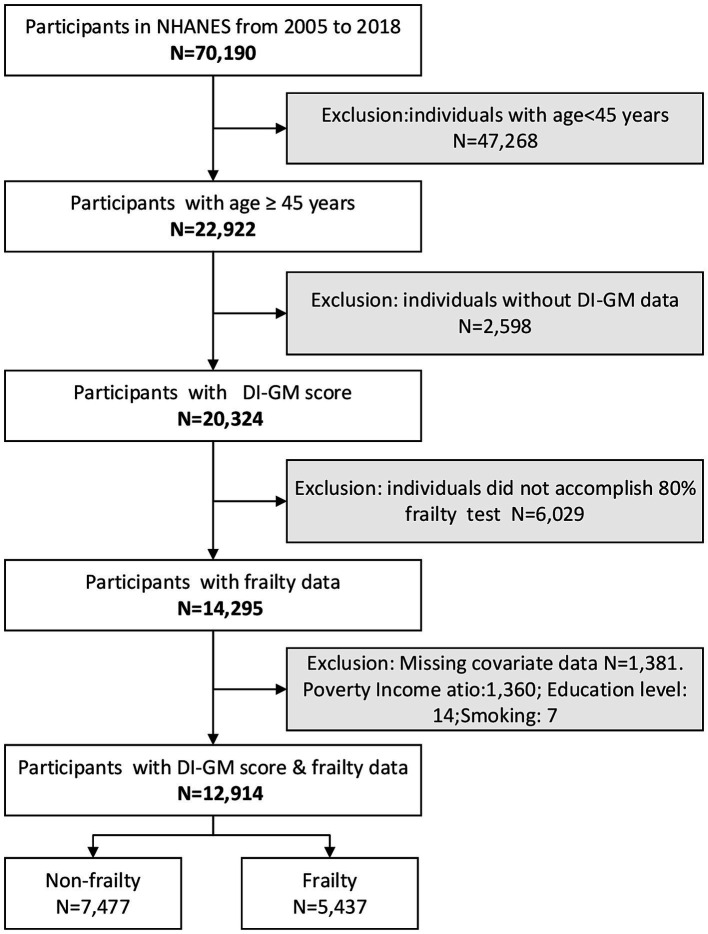
Flow diagram of the screening and enrollment of study participants. DI-GM, Dietary index for gut microbiota; NHANES, National Health and Nutrition Examination Survey.

The screening process is depicted in Figure 1.

The NHANES employs a multistage, stratified probability cluster sampling design to generate nationally representative estimates for the non-institutionalized U.S. civilian population. Data acquisition involves two sequential phases: (1) standardized computer-assisted personal interviews administered in households, followed by (2) detailed physiological measurements and laboratory tests conducted in Mobile Examination Centers (MECs), utilizing calibrated equipment and standardized protocols. The study protocol was approved by the National Center for Health Statistics Ethics Review Board, and all participants provided written informed consent. The NHANES data are publicly available at https://www.cdc.gov/nchs/nhanes/.

### 2.2 Valuation of DI-GM

Dietary exposure quantification in the NHANES was achieved via two sequentially administered 24-h dietary recall interviews, utilizing the validated Automated Multiple-Pass Method protocol. Initial interviews were conducted in person at MECs, with follow-up telephone recalls performed 3–10 days later to enhance temporal variability capture. DI-GM, developed by Kase et al. (12), comprises 14 dietary components: 10 beneficial for gut health (e.g., avocados, broccoli, chickpeas) and 4 detrimental (e.g., red meat, processed meats; Supplementary Table S2). Scoring criteria were defined as follows: For beneficial foods, 1 point was assigned if intake exceeded sex-specific median levels (0 points otherwise), whereas detrimental foods received 1 point if consumption fell below median thresholds (0 points otherwise). Individual scores were combined to yield the total DI-GM score, with higher values indicating healthier gut microbiota profiles. Based on NHANES data from 2005 to 2018, the scale for DI-GM scores extended from 0 to 10 and was categorized into quartiles based on population distribution: Q1 (≤3), Q2 (4), Q3 (5), and Q4 (≥6).

### 2.3 Definition of frailty

Frailty was evaluated through an index-based measurement, adhering to the standardized protocol established by Rockwood and coworker (21), which encompasses 49 variables across seven domains to capture multidimensional health deficits. This methodology was implemented as described by Jiang et al. (22), with the index calculated using variables spanning: cognition (1 item), dependency (16 items), depressive symptoms (7 items), comorbidities (13 items), hospital/nursing care utilization (5 items), anthropometrics (1 item), and laboratory parameters (6 items) (Supplementary Table S3). Participants failing to complete ≥ 80% of frailty-related items in the eligibility questionnaire were excluded. The frailty index was determined by aggregating the scores of these items and dividing them via the total count, resulting in a continuous value ranging from 0 (no deficits) to 1 (maximum deficits). For analytical purposes, this continuous variable was dichotomized into a binary frailty status: frailty was dichotomized into non-frail (≤0.21) and frail (>0.21) groups based on previous literature (23).

### 2.4 Covariates

Our analysis adopted several potential confounders as covariates, according to established literature and clinical rationale. These variables involved age, sex, race/ethnicity, marital status, educational level, poverty-to-income ratio (PIR), smoking status, alcohol use, and physical activity (12, 13, 19, 20, 24, 25).

Race was classified into five subgroups: Mexican American, Other Hispanic, Non-Hispanic White, Non-Hispanic Black, and Other Races. Educational attainment was categorized into three levels: less than high school, high school or equivalent, and college or higher. Smoking status was divided as never, current smoker and former smoker determined by responses to questionnaires, primarily the questions: “Have you smoked at least 100 cigarettes in your life?” and “Do you smoke now?.” Alcohol use was classified into three tiers: light (female: ≤2 cups/day; male: ≤1 cup/day), moderate (female: 2–4 cups/day; male: 1–3 cups/day), and heavy (female: ≥5 cups/day or ≥4 cups/time; male: ≥4 cups/day or ≥3 cups/time). Physical activity levels were categorized based on weekly metabolic equivalent (MET) values, calculated as follows: physical activity (MET-min/week) = MET × weekly frequency × duration per activity. Participants were stratified into four activity levels: sedentary (<600), insufficient (≤600 < 900), moderate (≤900 < 1,200), and vigorous (≥1,200) (13). For analytical purposes, age and PIR were modeled as continuous variables in logistic regression analyses. In descriptive and subgroup analyses, age was partitioned into ≤ 45 ≤ 60, < 60 ≤ 75, and ≥ 75 years, while PIR was divided into three categories: ≤ 1.3, < 1.30 ≤ 3.5, and > 3.5.

### 2.5 Statistical analysis

This study conducted a secondary analysis utilizing publicly accessible datasets, ensuring transparency and reproducibility. Distributions of the variables were examined using visual methods (histograms and Q–Q plots) as well as the Kolmogorov–Smirnov test. Chi-square tests were employed to evaluate intergroup differences for categorical variables, whereas Student test or one-way ANOVA was utilized for continuous data following a normal distribution. Multiple comparisons were conducted using either the Student–Newman–Keuls (SNK) or the least significant difference (LSD) methods. Categorical variables were expressed as frequency (*n*, %), with continuous variables summarized as mean ± standard deviation (SD) for normally distributed data. Logistic regression models were used to estimate odds ratios (ORs) and corresponding 95% confidence intervals for the associations between the included variables and frailty status.

To comprehensively evaluate the association between DI-GM and frailty risk, we first conducted univariate (Supplementary Table S4) and multivariable-adjusted binary logistic regression analyses. The multivariable analysis incorporated a hierarchical adjustment strategy across three sequential models: Model 1 (demographics: age, sex, ethnicity), Model 2 (socioeconomic factors: education, marital status, PIR), and Model 3 (behavioral confounders: alcohol, smoking, physical activity), with full estimates tabulated in Table 1. Sensitivity analyses retaining all eligible participants (*n* = 12,475) were performed to address outlier data assumptions. Nonlinear associations were assessed via restricted cubic spline (RCS) regression, while threshold effects and inflection points were identified using segmented logistic regression with Bootstrap resampling (1,000 iterations). Subgroup analyses stratified by age, sex, race/ethnicity, marital status, education, and income further evaluated effect heterogeneity, with interaction terms tested via likelihood ratio tests. To delineate mechanisms linking the DI-GM score to frailty, we developed a mediation model with BMI as the predefined mediator. Bootstrap resampling (1,000 iterations) was applied to minimize sampling variability, reinforcing inferential robustness. Mediation effect size was quantified as the indirect-to-total effect ratio (indirect effect proportion), expressed as a percentage for clinical interpretability. Furthermore, to minimize the impact of missing data on the results, we performed multiple imputation using chained equations, generating five complete datasets aligned with the variables specified in the final statistical model.

**Table 1:** Population characteristics by categories of DI-GM score (*n* = 12,914).

Variables		DI-GM score				*p* value
	Total	Q1 (0–3)	Q2 (4)	Q3 (5)	Q4 (≥6)	
	*n* = 12,914	(*n* = 939)	(*n* = 1805)	(*n* = 3,082)	(*n* = 7,088)	
Gender, *n* (%)						<0.001
Male	6,421 (49.7)	495 (52.7)	955 (52.9)	1,600 (51.9)	3,371 (47.6)	
Female	6,493 (50.3)	444 (47.3)	850 (47.1)	1,482 (48.1)	3,717 (52.4)	
Race, *n* (%)						<0.001
Mexican American	1,517 (11.7)	78 (8.3)	194 (10.7)	378 (12.3)	867 (12.2)	
Other Hispanic	1,128 (8.7)	57 (6.1)	138 (7.6)	274 (8.9)	659 (9.3)	
Non-Hispanic White	6,545 (50.7)	426 (45.4)	801 (44.4)	1,527 (49.5)	3,791 (53.5)	
Non-Hispanic Black	2,795 (21.6)	326 (34.7)	544 (30.1)	715 (23.2)	1,210 (17.1)	
Other race	929 (7.2)	52 (5.5)	128 (7.1)	188 (6.1)	561 (7.9)	
Age (y), *n* (%)						<0.001
≤ 45 ≤ 60	2,276 (17.6)	215 (22.9)	407 (22.5)	599 (19.4)	1,055 (14.9)	
< 60 ≤ 75	7,304 (56.6)	538 (57.3)	998 (55.3)	1,715 (55.6)	4,053 (57.2)	
> 75	3,334 (25.8)	186 (19.8)	400 (22.2)	768 (24.9)	1980 (27.9)	
Education level, *n* (%)						<0.001
Less than high school	3,830 (29.7)	311 (33.1)	625 (34.6)	1,007 (32.7)	1,887 (26.6)	
High school or equivalent	3,156 (24.4)	270 (28.8)	504 (27.9)	790 (25.6)	1,592 (22.5)	
College or above	5,928 (45.9)	358 (38.1)	676 (37.5)	1,285 (41.7)	3,609 (50.9)	
Marital status, *n* (%)						0.059
Married or lived with partners	7,334 (56.8)	521 (55.5)	978 (54.2)	1,754 (56.9)	4,081 (57.6)	
Living alone	5,580 (43.2)	418 (44.5)	827 (45.8)	1,328 (43.1)	3,007 (42.4)	
Poverty income ratio, *n* (%)						<0.001
≤ 1.30	4,175 (32.3)	345 (36.7)	713 (39.5)	1,080 (35)	2,037 (28.7)	
< 1.30 ≤ 3.5	5,252 (40.7)	388 (41.3)	745 (41.3)	1,255 (40.7)	2,864 (40.4)	
> 3.5	3,487 (27.0)	206 (21.9)	347 (19.2)	747 (24.2)	2,187 (30.9)	
BMI (kg/m^2^)	29.5 ± 6.3	30.5 ± 6.8	30.1 ± 6.7	29.8 ± 6.5	29.1 ± 6.0	<0.001
Frailty, *n* (%)						<0.001
Non-frailty	7,477 (57.9)	479 (51)	961 (53.2)	1,644 (53.3)	4,393 (62)	
Frailty	5,437 (42.1)	460 (49)	844 (46.8)	1,438 (46.7)	2,695 (38)	
Alcohol use, *n* (%)						<0.001
Light	4,420 (64.8)	273 (59.1)	515 (57.8)	972 (61.1)	2,660 (68.6)	
Moderate	1,296 (19.0)	96 (20.8)	188 (21.1)	298 (18.7)	714 (18.4)	
Heavy	1,104 (16.2)	93 (20.1)	188 (21.1)	321 (20.2)	502 (13)	
Smoke status, *n* (%)						<0.001
Never	5,979 (46.3)	405 (43.1)	743 (41.2)	1,391 (45.1)	3,440 (48.5)	
Current	4,752 (36.8)	343 (36.5)	652 (36.1)	1,067 (34.6)	2,690 (38)	
Former	2,183 (16.9)	191 (20.3)	410 (22.7)	624 (20.2)	958 (13.5)	
Physical activity, *n* (%)						<0.001
Sedentary	6,057 (46.9)	484 (51.5)	931 (51.6)	1,532 (49.7)	3,110 (43.9)	
Insufficient	1,065 (8.2)	73 (7.8)	126 (7)	235 (7.6)	631 (8.9)	
Moderate	1,439 (11.1)	103 (11)	187 (10.4)	320 (10.4)	829 (11.7)	
Vigorous	4,353 (33.7)	279 (29.7)	561 (31.1)	995 (32.3)	2,518 (35.5)	

Percentages for categorical variables. DI-GM, dietary index for gut microbiota; Q, quartile.

All statistical procedures were conducted using R software (v4.2.2; R Foundation, https://www.R-project.org), which employs R as the computational backbone and leverages Python for its streamlined graphical interface (26). Effect sizes and corresponding *p*-values were calculated, reported, and compared across all models, with statistical significance defined as a two-tailed *p*-value below 0.05.

## 3 Results

### 3.1 Features of the participants

Table 2 presents the demographic and clinical characteristics of 12,914 participants (49.7% male; 50.3% female) categorized into quartiles based on DI-GM scores. Participants in the highest DI-GM quartile (Q4) exhibited a significantly lower prevalence of frailty compared to those in the lowest quartile (Q1) (38% vs. 49%, respectively). Notably, participants in Q4 group demonstrated considerably greater educational attainment, along with a higher prevalence of female, non-Hispanic White, married status, less alcohol intake and tobacco denial.

**Table 2:** Association between DI-GM score and frailty odds ratio (*n* = 12,914).

DI-GM Score			Crude		Model 1		Model 2		Model 3	
	*n*. total	*n*. event %	OR (95% CI)	*p* value	Adj. OR (95% CI)	*p* value	Adj. OR (95% CI)	*p* value	Adj. OR (95% CI)	*p* value
Overall	12,914	5,437 (42.1)	0.86 (0.84–0.88)	<0.001	0.87 (0.85–0.89)	<0.001	0.9 (0.88–0.93)	<0.001	0.91 (0.88–0.95)	<0.001
Cut values										
Q1: 0–3	939	460 (49)	1 (Ref.)		1 (Ref.)		1 (Ref.)		1 (Ref.)	
Q2: 4	1805	844 (46.8)	0.91 (0.78–1.07)	0.267	0.92 (0.79–1.08)	0.324	0.89 (0.76–1.05)	0.163	0.86 (0.68–1.1)	0.23
Q3: 5	3,082	1,438 (46.7)	0.91 (0.79–1.05)	0.211	0.93 (0.8–1.08)	0.326	0.96 (0.82–1.11)	0.577	0.93 (0.74–1.16)	0.506
Q4: ≥6	7,088	2,695 (38)	0.64 (0.56–0.73)	<0.001	0.66 (0.58–0.76)	<0.001	0.74 (0.64–0.85)	<0.001	0.74 (0.6–0.92)	0.005
*p* for trend				<0.001		<0.001		<0.001		<0.001

(1) The DI-GM scores (range 0–10) were stratified into four categories based on clinical relevance: 0–3, 4, 5, and ≥ 6 groups.

(2) Abbreviations: DI-GM, dietary index for gut microbiota; overall, DI-GM was treated as a continuous variable; OR, odds ratio; CI, confidence interval; Ref, reference; crude, DI-GM was performed multivariate logistics regression without any covariate.

(3) Model 1, adjusted as adjusted for covariate gender, race and age; Model 2, adjusted as for Model 1, additionally adjusted for education level, marital status and poverty income ratio; Model 3, adjusted as for Model 3, adjusted as for Model 2, additionally adjusted for alcohol use, smoking status and physical activity.

In addition, comparison between excluded and included populations (Supplementary Table S1) revealed distinct characteristics: the excluded group exhibited a markedly higher proportion of individuals aged 45–60 years (71.6% vs. 17.6%) and demonstrated socioeconomic advantages, including elevated educational attainment (50.8% vs. 45.9%) and household income levels (PIR > 3.5: 40.7% vs. 27%). The observed disparities suggest that caution is warranted when generalizing the study findings to populations with higher socioeconomic status and educational attainment.

### 3.2 Relationship between DI-GM and frailty

**Figure 2:**
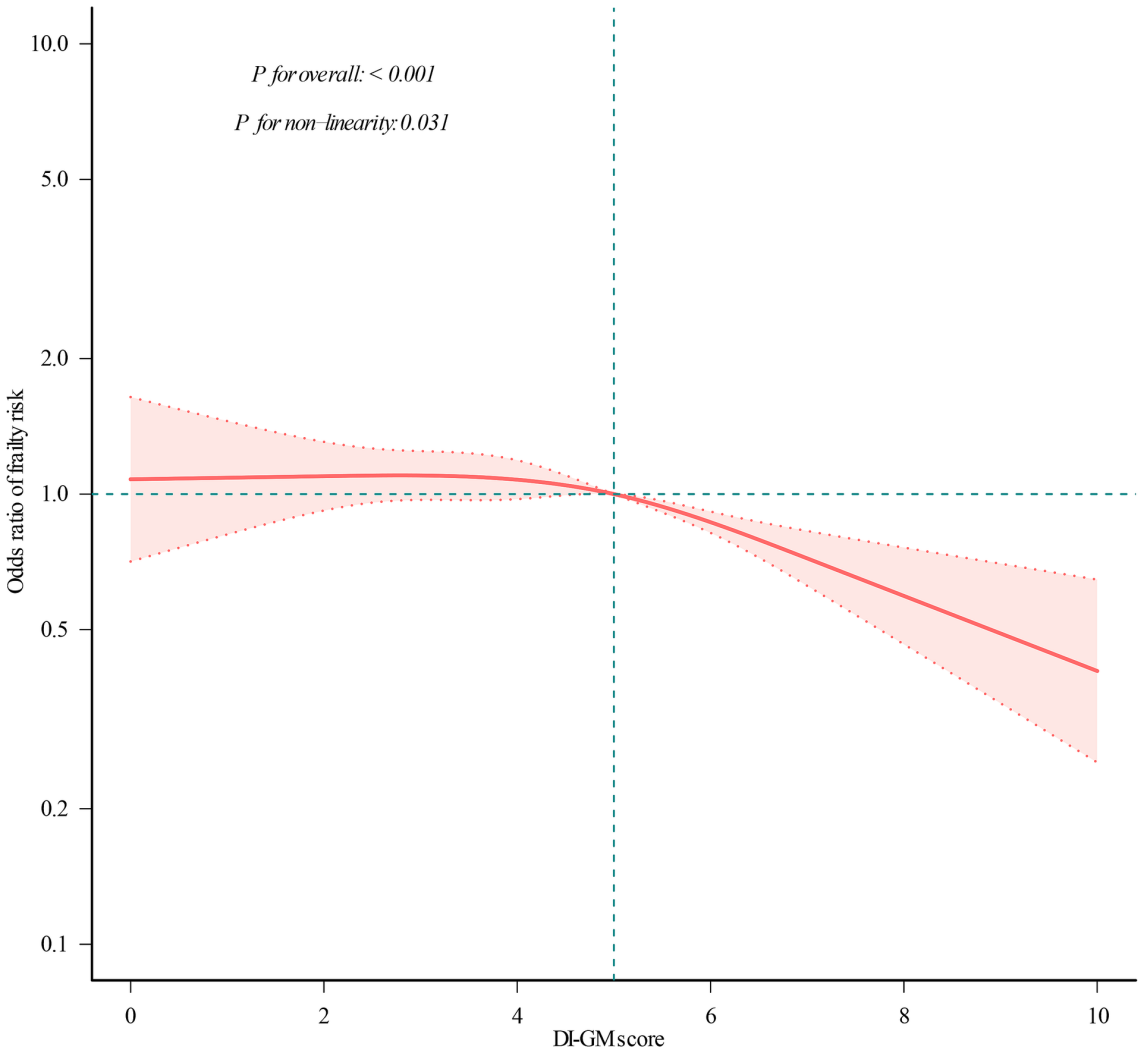
Restricted cubic spline analysis with multivariate-adjusted correlations between DI-GM score and frailty odds ratio. The red solid line indicates the expected values, and the dotted lines represent the 95% confidence intervals. They were adjusted for age, sex, marital status, race/ethnicity, educational attainment, household income, smoking status, physical activity, and alcohol use. All the data was presented.

Tables 1, 3, along with Figure 2 and Supplementary Table S5, examine the relationship between DI-GM scores and frailty risk by using different statistical models. Table 1 illustrates a consistent inverse relationship, showing that increased DI-GM scores are associated with a reduced risk of frailty. This trend is evident in the crude model, which reveals a strong negative correlation (OR = 0.86, 95% CI: 0.84–0.88, *p* < 0.001). The association remains robust across adjusted models (Model 1: OR = 0.87, Model 2: OR = 0.90, Model 3: OR = 0.91; all *p* < 0.001), reinforcing the reliability of this finding. Quadruple analysis further substantiates this pattern, with frailty risk progressively declining across quartiles and the most pronounced reduction observed in Q4 (Crude: OR = 0.64, Model 1: OR = 0.66, Model 2: OR = 0.74, Model 3: OR = 0.74; all *p* < 0.001).

**Table 3:** Threshold effect analysis of the association between DI-GM score and frailty risk.

Item	Breakpoint. OR (95%CI)	*p* value
E_BK1	4.082 (3.981, 4.183)	NA
slope1	0.999 (0.907–1.1)	0.983
slope2	0.874 (0.806–0.947)	0.001
Likelihood Ratio test		0.037

Abbreviations: OR, odds ratio; CI, confidence interval; DI-GM, dietary index for gut microbiota. Adjusted for age, sex, marital status, race/ethnicity, education level, family income, smoking status, physical activity and alcohol use 100% of the data is presented.

To evaluate the robustness of the DI-GM–frailty association against methodological assumptions, we excluded outliers for BMI and performed sensitivity analysis (*n* = 12,475). As summarized in Supplementary Table S4, consistent with results in Table 1, the inverse association persisted across all analytical models, with each 1-unit DI-GM increase conferring a 9% frailty risk reduction in the fully adjusted model (Model 3: OR = 0.91, 95% CI = 0.88–0.95, *p* < 0.001). Quartile-stratified analyses revealed a monotonic dose–response relationship (*p* for trend < 0.01), where participants in the highest DI-GM quartile (Q4: DI-GM ≥ 6) exhibited a 26% lower frailty risk compared to Q1 (OR = 0.74, 95% CI = 0.59–0.92, *p* < 0.01). Collectively, these findings demonstrate a robust inverse association between DI-GM scores and frailty risk, characterized by both dose-dependent, as evidenced in Table 1 and Supplementary Table S4.

To further investigate the DI-GM–frailty relationship, we conducted RCS regression. As illustrated in Figure 2, a significant nonlinear association was observed (*p* for nonlinearity = 0.031), characterized by a curvilinear dose–response pattern.

Subsequent threshold analysis (Table 3) identified a critical inflection point at DI-GM = 4.082 (95% CI: 3.98–4.18), indicating a transition between low-risk and high-risk regimes. Below this value, frailty risk remains stable (slope 1: OR = 0.999, *p* = 0.983), indicating little influence of DI-GM in this range. Conversely, beyond this point, each 1-unit increase in DI-GM score was associated with a 12.6% reduction in frailty risk (OR = 0.874; 95% CI: 0.806–0.947). The statistical relevance of this threshold effect is further confirmed by the likelihood ratio test (*p* = 0.037), validating the presence of a critical DI-GM threshold in frailty prevention. This non-linearity suggests that the protective association of DI-GM scores may be heterogeneous across its range, with enhanced effects at higher level (DI-GM > 4.082).

### 3.3 Subgroup and interaction analysis

**Figure 3:**
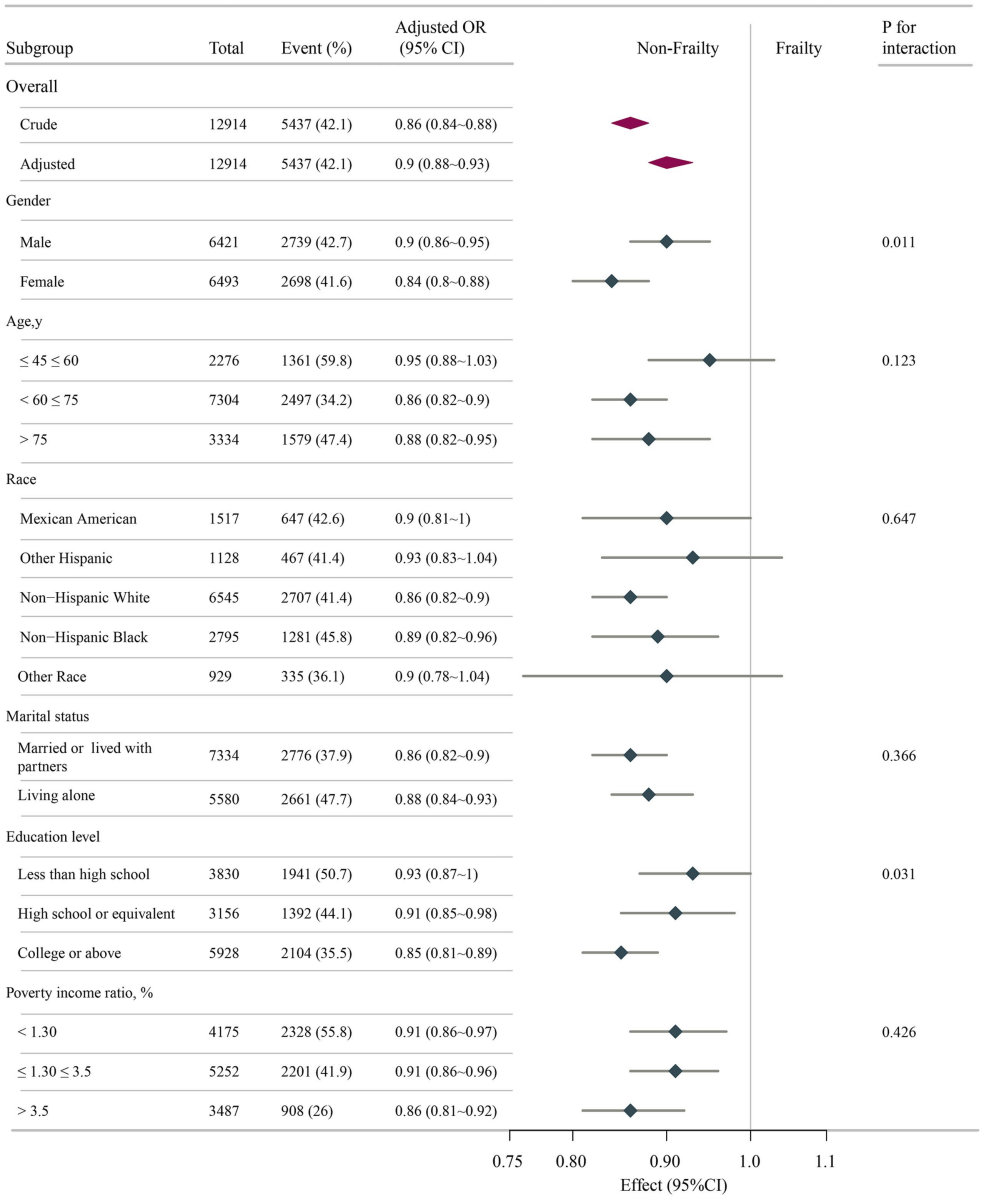
Associations between DI-GM score and frailty odds ratio in different subgroups. All stratification factors, except for the stratification component itself, were adjusted for variables in Model 3. DI-GM, dietary index for gut microbiota; OR, odds ratio; CI, confidence interval.

To further evaluate possible interactions in the association between DI-GM scores and frailty risk, subgroup analyses were conducted across many variables. Figure 3 confirms a consistent inverse relationship across subgroups, with no significant interactions for marital status (*p* = 0.366), race (*p* = 0.647), or income (*p* = 0.426). Notably, sex (*p* = 0.011) and education attainment (*p* = 0.031) modified the effect, showing a stronger protective association in females (OR = 0.84) vs. males (OR = 0.90) and in those with higher education (OR = 0.85) vs. lower education (OR = 0.93). DI-GM showed significant gender/education-level interactions in frailty associations (*p*-interaction < 0.05), yet maintained robust inverse trends consistently across all subgroups.

### 3.4 Mediation analysis

**Figure 4:**
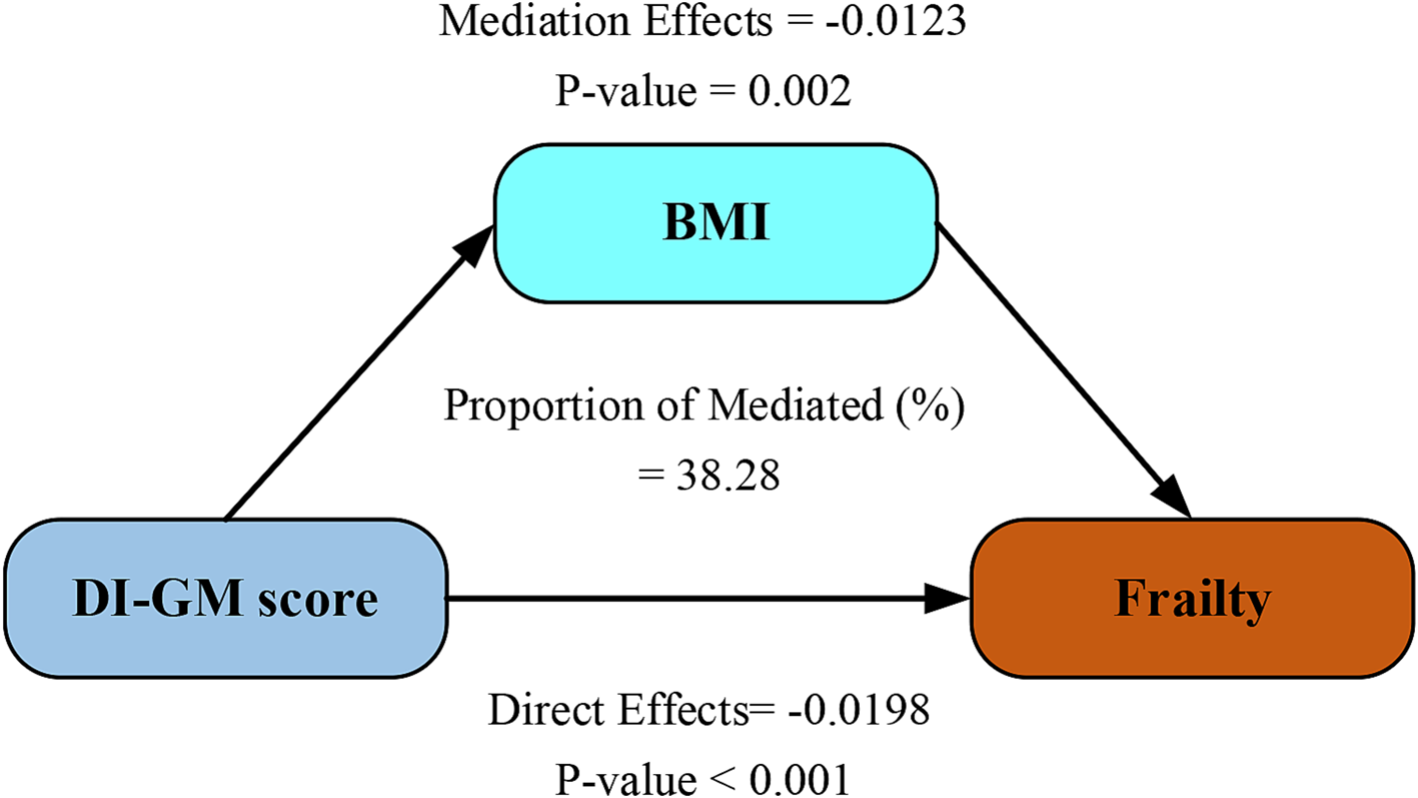
Mediation analysis of BMI in the relationship between DI-GM score and frailty. Models were adjusted for age, sex, ethnicity, marital status, education attainment, PIR level, physical activity, smoking and drinking status. DI-GM, dietary index for gut microbiota; BMI, body mass index.

As demonstrated in Figure 4, BMI significantly mediated 38.28% of the total effect of DI-GM score on frailty (95% CI: 26.62–62.60; *p* < 0.001) after adjustment for demographic, behavioral, and socioeconomic confounders. This finding highlights BMI as a clinically relevant mechanistic pathway linking genetic susceptibility to frailty progression.

## 4 Discussion

This cross-sectional study examined the associations between DI-GM and frailty in U.S. adults aged 45 years and older. Analyses revealed a significant nonlinear relationship, with a threshold effect at a DI-GM score of 4.082. Higher DI-GM scores were inversely associated with frailty risk, notably among women and individuals with higher educational attainment. BMI demonstrated partial mediation (38.28% of total effect) between DI-GM exposure and frailty phenotypes. Our threshold analysis (DI-GM ≥ 4.082) identifies an actionable dietary benchmark, offering a scalable strategy for healthy aging.

Unlike traditional dietary indices (e.g., Mediterranean Diet Score) that evaluate general dietary quality, DI-GM pioneers a microbiota-centric approach by quantifying foods that directly modulate gut ecosystem functionality, thereby enabling targeted frailty prevention strategies. It emphasizes the intake of dietary fiber and fermented foods, which promote gut microbiota diversity and the production of anti-inflammatory metabolites, such as short-chain fatty acids (SCFAs) (27). Dietary fiber intake classified as beneficial for gut microbiota under DI-GM criteria is associated with the abundance of SCFA-producing bacterial genera, whereas long-term low-fiber diets may lead to intergenerational declines in microbial diversity (28, 29). Conversely, high–fat/high–sugar diets promote the proliferation of conditional pathogens like Proteobacteria while reducing the Firmicutes–to–Bacteroidetes ratio, a dysbiotic state closely linked to gut barrier dysfunction (30, 31). Clinically, the Mediterranean diet—rich in polyphenols and omega-3 fatty acids—has been shown to significantly enhance microbiota diversity indices and foster colonization of anti-inflammatory genera such as Bifidobacterium and Lactobacillus (32).

Frail individuals commonly exhibit marked reductions in gut microbiota alpha diversity (e.g., Shannon index). For instance, species richness in frail older adults decreases by 20–30% compared to healthy controls, and this loss of diversity inversely correlates with elevated systemic inflammatory markers (e.g., Interleukin-6, C-reactive protein) (33, 34). These findings suggest that dietary patterns regulate the “microbiota–gut–frailty” axis by modulating microbial diversity. Sustained intake of prebiotic-rich foods is necessary to maintain anti-inflammatory metabolite levels, thereby mitigating sarcopenia and chronic inflammation (35). This study identified a nonlinear association between improvements in DI-GM score and frailty risk (Figure 3). Notably, frailty risk demonstrated a measurable decline only when DI-GM scores surpassed a critical threshold of 4.082. This threshold-dependent phenomenon delineates a significant dose–response pattern, characterized by minimal risk modulation below the cutoff and a progressive protective effect beyond it. These findings, in conjunction with previous research, provide robust empirical support for the crucial regulatory function of gut microbiota in attenuating frailty progression through metabolic pathway modulation.

To investigate the potential mechanism by which DI-GM score improvement reduces frailty risk, we conducted mediation analyses (Figure 4), identifying BMI as a statistically significant mediator in the DI-GM-frailty association (mediation effect: 38.28%). This finding aligns with established epidemiological evidence demonstrating BMI’s critical role in frailty pathogenesis. Jayanama et al. established that elevated BMI (>25.0 kg/m^2^) predicts heightened frailty indices in both cross-sectional evaluations and longitudinal observations (36). Sun’s longitudinal BMI trajectory modeling identified sustained high adiposity patterns as conferring greater frailty risk compared to stable normal-weight profiles (37). Yuan’s systematic review and meta-analysis, including community-dwelling older adults aged ≥60 years, further confirmed a strong positive correlation between BMI and frailty risk (38). Chronic low-grade inflammatory responses mediated by adiposity constitute a central pathophysiological mechanism linking elevated BMI to frailty susceptibility (39). Growing evidence indicates gut-microbiota-targeting diets regulate immune function and influence disease pathways, including frailty development (40, 41). These interactions underscore complex links between metabolic status, inflammatory processes, gut health, and frailty progression. The bidirectional interplay between BMI and gut microbiota operates through three principal mechanistic axes: energy homeostasis regulation, inflammatory modulation, and intestinal barrier maintenance. Individuals with elevated BMI (overweight/obese) exhibit gut dysbiosis characterized by reduced alpha-diversity, increased Firmicutes/Bacteroidetes ratio, depletion of SCFA-producing bacteria, and enrichment of pro-inflammatory species (42, 43). Obesity-associated microbial consortia, particularly Firmicutes-dominated clusters, upregulate carbohydrate-active enzyme expression, thereby augmenting energy absorption and adipocyte lipid accumulation (44). Conversely, microbial metabolites orchestrate systemic energy balance through multi-organ crosstalk: (1) butyrate enhances intestinal barrier integrity via tight junction protein upregulation, (2) inhibits peroxisome proliferator-activated receptor gamma-mediated adipogenesis, and (3) stimulates glucagon-like peptide-1 secretion to regulate glucose-lipid homeostasis (45). Lipopolysaccharide translocation triggered by dysbiosis induces adipose tissue inflammation and suppresses lipolysis through Toll-like receptor 4 signaling pathway activation (46). Our mediation analysis identifies BMI as a critical modulator in the microbiota-frailty axis, suggesting novel therapeutic targets at this metabolic interface. Clinically, managing frail patients requires combining tailored diets to modify gut bacteria, routine BMI checks using body scans, and gut microbiome testing to assess frailty risks.

Subgroup analyses revealed a pronounced association of DI-GM against frailty, particularly evident in female participants and those with advanced educational attainment. The augmented protective association observed in females may arise from dynamic hormone–microbiome interplay. Estrogen-mediated reinforcement of intestinal barrier integrity and promotion of Lactobacillus colonization appear to act synergistically with DI-GM to potentiate SCFAs biosynthesis (47). The postmenopausal estrogen declines precipitate gut dysbiosis, potentially accounting for diminished protective effects (OR = 0.88) in elderly females (>75 years subgroup), despite comparable DI-GM profiles. Notably, dietary analyses demonstrated greater consumption of fiber-rich and fermented foods among females compared to males (48), correlating with their disproportionate representation in the highest DI-GM quartile (Q4: 54.2% vs. 45.8%). These nutritional patterns appear conducive to maintaining keystone commensals such as Bifidobacterium, organisms critically involved in folate biosynthesis and DNA methylation homeostasis. Although current studies confirm significant estrogen-microbiota correlations (49, 50), NHANES database limitations impede mechanistic interpretation of estrogen’s mediation in DI-GM-conferred frailty protection. Key constraints include undocumented menopausal status (natural/surgical), incomplete estrogenic biomarker profiling (cycle-specific estrone/estradiol measurements), and absent hormone replacement therapy records. Thus, longitudinal estrogen dynamics monitoring is essential to delineate precise mechanisms.

The socioeconomic gradient in DI-GM efficacy manifested through enhanced protection against frailty in highly educated individuals (OR = 0.85 vs. 0.93 in lower education strata). Mechanistically, advanced education confers greater protective benefits through multidimensional pathways: Health literacy enables a nuanced understanding of nutritional guidelines, driving preferential selection of prebiotic-rich foods (whole grains, legumes) that optimize DI-GM adherence (51). Educational attainment further correlates with dietary diversity and consistent intake of methyl donors (folate, vitamin B12), serving as critical substrates for DNA methylation processes (52). Recent epidemiological evidence demonstrates education–associated hypomethylation at the IL-10 promoter region, potentiating anti-inflammatory cytokine production—an effect amplified in subjects maintaining microbiota–supportive dietary patterns (53). These findings consist with our observations, suggesting that education may establish an epigenetically “primed state” that facilitates the translation of dietary inputs (e.g., DI-GM) into health benefits, potentially mediated through microbiota–epigenome interaction. This dual-axis interaction demonstrates the convergence of biological determinants (e.g., hormonal regulation, epigenetic modulation) with social determinants (e.g., health literacy), offering pivotal implications for advancing clinically actionable precision nutrition strategies.

Our findings highlight the translational potential of DI-GM as a public health tool. Specifically, the identified threshold (DI-GM ≥ 4.082) could inform national dietary guidelines targeting older adults. For example: (1) Community-level interventions: Subsidizing fiber-rich foods (e.g., whole grains, legumes) in low-income neighborhoods to reduce socioeconomic disparities in DI-GM adherence. (2) Clinical integration: Incorporating DI-GM screening into geriatric assessments to identify high-risk individuals (DI-GM < 4) for tailored nutritional counseling. 3) Policy advocacy: Collaborating with agricultural sectors to increase the production of fermented foods (e.g., yogurt, kimchi), which are key DI-GM components linked to gut health (32, 34). These strategies align with the WHO’s “Decade of Healthy Aging” framework, emphasizing cost-effective dietary interventions to mitigate frailty burden (2).

While our investigation demonstrates methodological rigor, several limitations warrant consideration. First, the cross-sectional design of the NHANES database restricts causal inference between diet-microbiota patterns and frailty development. Future research needs longitudinal cohorts with repeated dietary assessments, microbial monitoring, and frailty tracking to clarify temporal relationships among multifactorial influences. Second, 24-h dietary recalls present inherent constraints: (1) recall bias, particularly in older adults with cognitive decline; (2) inadequate capture of daily/weekly dietary variations, potentially underestimating long-term microbiome impacts on gut microbiota and frailty risk (54, 55); and (3) discrepancies between reported intake and actual microbial nutrient exposure (e.g., lactase activity differences). Combining food biomarkers (plasma carotenoids, urinary nitrogen) with metagenomic metrics (polysaccharide utilization genes) could improve nutritional assessments (55). Third, the ethnogeographic limitations of NHANES data—particularly the absence of validation in Asian/European cohorts—substantially restrict the external validity of these findings. Inadequate gut microbiota sampling frequency (Fecal sample collection was exclusively conducted in 2013–2014 cycle) further restricts understanding of host–microbe interactions. Future DI-GM validation requires: (1) Multi-ethnic replication using UK Biobank’s diverse cohorts; (2) Multi-omics mapping integrating fecal metagenomics, SCFA metabolomics, and single-cell transcriptomics for microbial functional profiling; (3) Germ-free model interventions establishing diet-microbiota dose–response relationships. Fourth, though we adjusted for key confounders, residual confounding from unmeasured covariates (e.g., socioeconomic mobility and environmental exposures) remains plausible.

## 5 Conclusion

Our findings reveal a robust inverse association between elevated DI-GM scores and frailty risk, with demographic-specific patterns showing special efficacy in females and individuals with advanced educational attainment. Mediation analyses further demonstrated a significant indirect pathway, with BMI partially explaining the association between DI-GM scores and frailty status. The identification of a clinically relevant threshold effect (DI-GM = 4.082) underscores the public health importance of attaining dietary benchmarks to optimize frailty prevention. These findings collectively highlight the translational potential of microbiota–targeted nutritional frameworks as geroprotective strategies, particularly for modulating gut microbial diversity in aging populations.

## Data Availability

The datasets analyzed in this study are publicly available from the NHANES database, managed by the Centers for Disease Control and Prevention. All data can be accessed through the official NHANES website. The specific datasets used in this analysis are from the survey cycles 2005–2018.
