# Supplementary material for: Association of dietary index for gut microbiota with frailty in middle-aged and older Americans: a cross-sectional study and mediation analysis
Source: Front Nutr. 2025 Jun 25;12:1615386. doi: 10.3389/fnut.2025.1615386 (PMC12237688; doi:10.3389/fnut.2025.1615386)
Supplement: Supplementary file 1 [file Data_Sheet_1.pdf]

Supplementary Material

To: **Association of Dietary Index for Gut Microbiota with Frailty in Middle-Aged and Older Americans: A Cross - sectional Study and Mediation Analysis**

Xiaodan Li, Ying Liu, Chuyi Shen, Changzhuan Shao, and Hongke Jiang

1 Supplementary Table S1

Supplementary Table S1. Comparison of baseline characteristics between included and excluded populations

Variables	Total	Excluded	Included	p
	(n = 22,922)	(n = 10,008)	(n = 12,914)	
**Gender, n (%)**				0.032
Male	11254 (49.1)	4833 (48.3)	6421 (49.7)	
Female	11668 (50.9)	5175 (51.7)	6493 (50.3)	
**Race, n (%)**				< 0.001
Mexican American	3083 (13.4)	1566 (15.6)	1517 (11.7)	
Other Hispanic	2200 (9.6)	1072 (10.7)	1128 (8.7)	
Non-Hispanic White	10306 (45.0)	3761 (37.6)	6545 (50.7)	
Non-Hispanic Black	5036 (22.0)	2241 (22.4)	2795 (21.6)	
Other Race	2297 (10.0)	1368 (13.7)	929 (7.2)	
**Age (y), n (%)**				< 0.001
≤ 45 ≤ 60	9442 (41.2)	7166 (71.6)	2276 (17.6)	
< 60 ≤ 75	8968 (39.1)	1664 (16.6)	7304 (56.6)	
> 75	4512 (19.7)	1178 (11.8)	3334 (25.8)	
**Marital status, n (%)**				< 0.001
Married or lived with partners	13675 (59.7)	6341 (63.4)	7334 (56.8)	
Living alone	9247 (40.3)	3667 (36.6)	5580 (43.2)	
**Education level, n (%)**				< 0.001
Less than high school	6564 (28.7)	2734 (27.4)	3830 (29.7)	
High school or equivalent	5323 (23.3)	2167 (21.8)	3156 (24.4)	
College or above	10988 (48.0)	5060 (50.8)	5928 (45.9)	
**Poverty Income Ratio, n (%)**				< 0.001
≤ 1.30	6014 (29.3)	1839 (24.2)	4175 (32.3)	
< 1.30 ≤ 3.5	7921 (38.6)	2669 (35.1)	5252 (40.7)	
> 3.5	6579 (32.1)	3092 (40.7)	3487 (27)	
**Smoke Status, n (%)**				< 0.001
Never	11632 (50.8)	5653 (56.6)	5979 (46.3)	
Current	7243 (31.6)	2491 (24.9)	4752 (36.8)	
Former	4025 (17.6)	1842 (18.4)	2183 (16.9)	
**Alcohol Use, n (%)**				< 0.001
Light	3063 (15.9)	1147 (15.3)	1916 (16.3)	
Moderate	4292 (22.3)	1250 (16.6)	3042 (25.8)	
Heavy	11927 (61.9)	5115 (68.1)	6812 (57.9)	
**Physical activity, n (%)**				< 0.001
Insufficient	5625 (37.2)	2367 (34)	3258 (39.9)	
Moderate	955 (6.3)	396 (5.7)	559 (6.8)	
Vigorous	8544 (56.5)	4191 (60.3)	4353 (53.3)	
**BMI (kg/m^2)**	29.4 ± 6.7	29.2 ± 6.5	29.6 ± 6.8	< 0.001

2 Supplementary Table S2

Supplementary Table S2. Components and scoring calculation of DI-GM in National Health and Nutrition Examination Survey

Components	Food items	Criteria
**Beneficial food**	1. Avocados<br>2. Fermented dairy<br>3. Chickpeas<br>4. Coffee<br>5. Cranberries<br>6. Broccoli<br>7. Fiber<br>8. Soy milk<br>9. Tofu<br>10. Whole grains	**Score 1**: Consumption ⩾sex median<br>**Score 0**: Otherwise
**Unfavorable food**	11. Refined grains<br>12. Processed meat<br>13. Red meat	**Score 0** – Consumption ⩾ sex median<br>**Score 1** - Otherwise
	14. High-fat diet (% energy)	**Score 0** - Consumption⩾40%<br>**Score 1** - Otherwise

1) Abbreviations: DI-GM, dietary index for gut microbiota

2) DI-GM, developed by Bezawit E. Kase et al. (1, 2), comprises 14 dietary components: 10 beneficial for gut health (e.g., avocados, broccoli, chickpeas) and 4 detrimental (e.g., red meat, processed meats). The detailed items and theirs corresponding scoring criteria were shown in Table 1.

3 Supplementary Table S3

Supplementary Table S3. Evaluation of the frailty index/score

Items	scores
1.experience confusion/memory problems	yes=1, no=0
2.managing money difficulty	no difficulty=0, Some difficulty=0.33, much difficulty=0.66, unable to do=1
3.walking for a quarter mile difficulty	no difficulty=0, Some difficulty=0.33, much difficulty=0.66, unable to do=1
4.walking up ten steps difficulty	no difficulty=0, Some difficulty=0.33, much difficulty=0.66, unable to do=1
5.stooping, crouching, kneeling difficulty	no difficulty=0, Some difficulty=0.33, much difficulty=0.66, unable to do=1
6.lifting or carrying difficulty	no difficulty=0, Some difficulty=0.33, much difficulty=0.66, unable to do=1
7.house chore difficulty	no difficulty=0, Some difficulty=0.33, much difficulty=0.66, unable to do=1

8.preparing meals difficulty	no difficulty=0, Some difficulty=0.33, much difficulty=0.66, unable to do=1
9.standing up from armless chair difficulty	no difficulty=0, Some difficulty=0.33, much difficulty=0.66, unable to do=1
10.getting in and out of bed difficulty	no difficulty=0, Some difficulty=0.33, much difficulty=0.66, unable to do=1
11.using fork, knife, drinking from cup difficulty	no difficulty=0, Some difficulty=0.33, much difficulty=0.66, unable to do=1
12.dressing yourself difficulty	no difficulty=0, Some difficulty=0.33, much difficulty=0.66, unable to do=1
13.standing for long periods difficulty	no difficulty=0, Some difficulty=0.33, much difficulty=0.66, unable to do=1
14.grasp/holding small objects difficulty	no difficulty=0, Some difficulty=0.33, much difficulty=0.66, unable to do=1
15.attending social event difficulty	no difficulty=0, Some difficulty=0.33, much difficulty=0.66, unable to do=1
16.leisure activity at home difficulty	no difficulty=0, Some difficulty=0.33, much difficulty=0.66, unable to do=1
17.push or pull large objects difficulty	no difficulty=0, Some difficulty=0.33, much difficulty=0.66, unable to do=1
18.have little interest in doing things	nearly every day = 1, more than half the days = 0.66, several days = 0.33, no =0
19.feeling down, depressed, or hopeless	nearly every day = 1, more than half the days = 0.66, several days = 0.33, no =0
20.trouble sleeping or sleeping too much	nearly every day = 1, more than half the days = 0.66, several days = 0.33, no =0
21.feeling tired or having little energy	nearly every day = 1, more than half the days = 0.66, several days = 0.33, no =0
22.poor appetite or overeating	nearly every day = 1, more than half the days = 0.66, several days = 0.33, no =0
23.feeling bad about yourself	nearly every day = 1, more than half the days = 0.66, several days = 0.33, no =0
24.trouble concentrating on things	nearly every day = 1, more than half the days = 0.66, several days = 0.33, no =0
25.doctor ever said you had arthritis	yes = 1, no = 0
26.ever told you had thyroid problem	yes = 1, no = 0
27.ever told you had chronic bronchitis	yes = 1, no = 0
28.ever told you had cancer or malignancy	yes = 1, no = 0
29.ever told had congestive heart failure	yes = 1, no = 0
30.ever told you had coronary heart disease	yes = 1, no = 0
31.ever told you had angina/angina pectoris	yes = 1, no = 0
32.ever told you had heart attack	yes = 1, no = 0
33.ever told you had a stroke	yes = 1, no = 0
34.ever told you had high blood pressure	yes = 1, no = 0
35.doctor told you have diabetes	yes = 1, borderline=0.5, no =0
36.ever told you had weak/failing kidneys	yes = 1, no =0
37.urine leakage bother you?	greatly = 1, very much =0.75, somewhat= 0.5, only a little = 0.25, no=0
38.general health condition	excellent, very good, good = 0, fair, poor = 1
39.health now compared with 1 year ago	worse =1, better = 0
40.overnight hospital patient in last year	yes =1, no =0
41.times receive healthcare over past year	no=0, 1-4=0.5, ≥5 =1
42.number of prescription medicines taken	no =0, 1-4=0.5, ≥5 =1
43.body mass index (kg/m^2)	<18.5, ≥30=1；≥25, <30=0.5；≥18.5, ＜25=0

44.glycohemoglobin (%)	0%-5.7%=0, >5.7%=1
45.red blood cell count (million cells/ul)	M: ≥4.7, <6.1=0, Other=1；F: ≥4.2, ＜5.4=0, Other =1
46.hemoglobin (g/dl)	M: ≥13.5, <18 =0, Other =1；F: ≥12, <16 =0, Other=1
47.red cell distribution width (%)	≥11.6, <14.6=0, Other=1
48.lymphocyte percent (%)	≥20, <40=0, Other=1
49.segmented neutrophils percent (%)	≥40, <80=0, Other=1

The frailty index (FI) was operationalized following Jiang et al.'s methodology (3), comprising 49 items across seven functional domains:

1. **Cognition** (1 item),
2. **Dependency** (16 items),
3. **Depressive symptoms** (7 items),
4. **Comorbidities** (13 items),
5. **Healthcare utilization** (5 items),
6. **Anthropometrics** (1 item),
7. **Laboratory biomarkers** (6 items).

Data sources were categorized as:

- **Questionnaire-derived**: Cognition, Dependency, Depressive symptoms, Comorbidities, Healthcare utilization (NHANES interview modules);
- **Clinical measurements**: Anthropometrics (physical examination);
- **Objective biomarkers**: Laboratory parameters (blood/urine assays).

FI was calculated as the ratio of total score present to total items (FI =Σdeficits/49) and dichotomized into non-frailty (FI ≤ 0.21) versus frailty (FI > 0.21) per Hakeem et al.'s threshold (4).

4 Supplementary Table S4

Supplementary Table S4. Association of covariates and frailty odds ratio

**Variable**	**OR (95 CI)**	***P* value**
Gender: Male vs Female	0.96 (0.89~1.02)	0.204
Age, y (cont. var.)	0.99 (0.99~1)	<0.001
Race		
ref= Mexican American		
Other Hispanic	0.95 (0.81~1.11)	0.52
Non-Hispanic White	0.95 (0.85~1.06)	0.358
Non-Hispanic Black	1.14 (1~1.29)	0.045
Other Race	0.76 (0.64~0.9)	0.001
Education level		
ref= Less than high school		

High school or equivalent	0.77 (0.7~0.84)	<0.001	
College or above	0.54 (0.49~0.58)	<0.001	
Marital status			
Living alone vs Married or lived with partner	1.5 (1.39~1.61)	<0.001	
BMI (kg/m^2)	1.08 (1.08~1.09)	<0.001	
Poverty Income			
Ratio ref = ≤1.30			
≤ 1.30 ≤ 3.5	0.57 (0.53~0.62)	<0.001	
> 3.5	0.28 (0.25~0.31)	<0.001	
Alcohol Use			
ref = Light			
Moderate	1.02 (0.9~1.17)		0.725
Heavy	1.6 (1.4~1.83)	<0.001	
Smoke Status			
ref = Never			
Current	1.39 (1.28~1.5)	<0.001	
Former	1.88 (1.7~2.07)	<0.001	
Physical activity			
ref = Sedentary			
Insufficient	0.55 (0.48~0.62)	<0.001	
Moderate	0.4 (0.35~0.45)	<0.001	
Vigorous	0.38 (0.35~0.42)	<0.001	
DI-GM score (cont. var.)	0.86 (0.84~0.88)	<0.001	

DI-GM, Dietary index for gut microbiota; OR, odds ratio; CI, confidence interval. ref, reference. Table 3 presents odds ratios (OR) and confidence intervals (95% CI) for frailty risk factors.

1) Gender showed no significant association (OR = 0.96, p = 0.204). 2) Older adults faced higher frailty risk, with those aged 45–60 (OR = 0.35, p < 0.001) and 60–75 (OR = 0.60, p < 0.001) at lower risk than > 75 years. 3) Non–Hispanic Black individuals had increased vulnerability (OR = 1.14, p = 0.045), while higher education (OR = 0.54) and income (OR = 0.28) were protective. 4) Living alone (OR = 1.5), heavy alcohol use (OR = 1.6), and smoking (OR = 1.39–1.88) elevated risk, whereas vigorous exercise (OR = 0.38) and a higher DI-GM score (OR = 0.86) reduced frailty odds. 5) Higher BMI was associated with higher risk of frailty (OR = 1.08, CI:1.08~1.09, p < 0.001).

5 Supplementary Table S5

Supplementary Tables S5. Association between DI-GM score and frailty odds ratio (n = 12,475)

Variable	n. total	n. event_%	Crude		Model 1		Model 2		Model 3	
			OR (95%CI)	P value	adj. OR (95%CI)	adj.P value	adj. OR (95%CI)	adj.P value	adj. OR (95%CI)	adj.P value
Overall	12475	5138 (41.2)	0.86 (0.84~0.88)	<0.001	0.87 (0.85~0.89)	<0.001	0.9 (0.88~0.93)	<0.001	0.91 (0.88~0.95)	<0.001
DI_GM quantile										
Q1: 0-3	900	435 (48.3)	1(Ref)		1(Ref)		1(Ref)		1(Ref)	
Q2: 4	1745	809 (46.4)	0.92 (0.79~1.09)	0.336	0.92 (0.78~1.08)	0.31	0.89 (0.76~1.06)	0.195	0.86 (0.67~1.1)	0.228
Q3: 5	2942	1337 (45.4)	0.89 (0.77~1.03)	0.128	0.91 (0.78~1.06)	0.208	0.94 (0.8~1.1)	0.409	0.91 (0.73~1.15)	0.442
Q4: ⩾6	6888	2557 (37.1)	0.63 (0.55~0.73)	<0.001	0.66 (0.57~0.77)	<0.001	0.73 (0.63~0.85)	<0.001	0.74 (0.59~0.92)	0.006
P for Trend				<0.001		<0.001		<0.001		<0.001

1) The DI-GM scores (range 0 – 10) were stratified into four categories based on clinical relevance: 0 – 3, 4, 5, and ≥ 6 groups.

2) Abbreviations: DI-GM, Dietary index for gut microbiota; Overall, DI-GM was treated as a continuous variable; OR, odds ratio; CI, confidence interval; Ref, reference; Crude, DI-GM was performed multivariate logistics regression without any covariate.

3) Model 1, adjusted as adjusted for covariate gender, race and age; Model 2, adjusted as for Model 1, additionally adjusted for education level, marital status and poverty income ratio; Model 3, adjusted as for Model 3, adjusted as for Model 2, additionally adjusted for BMI, alcohol use, smoking status and physical activity.

References:

1. Kase BE, Liese AD, Zhang J, Murphy EA, Zhao L, Steck SE. The development and evaluation of a literature-based dietary index for gut microbiota. *Nutrients*. (2024) 16. doi: 10.3390/nu16071045

2. Zhang X, Yang Q, Huang J, Lin H, Luo N, Tang H. Association of the newly proposed dietary index for gut microbiota and depression: the mediation effect of phenotypic age and body mass index. *Eur Arch Psychiatry Clin Neurosci*. (2024). doi: 10.1007/s00406-024-01912-x

3. Jiang Z, Wang J, Cai X, Wang P, Liu S. L-shaped association of serum alpha-klotho and frailty among the middle-aged and older adults: results from NHANES 2007-2016. *BMC Geriatr*. (2023) 23: 716. doi: 10.1186/s12877-023-04324-z

4. Hakeem FF, Bernabe E, Sabbah W. Association between oral health and frailty among american older adults. *J Am Med Dir Assoc*. (2021) 22: 559-63. doi: 10.1016/j.jamda.2020.07.023
